# Hospitalization burden in children on dialysis: insights from the Italian Registry of Pediatric Chronic Dialysis (IRPCD)

**DOI:** 10.1007/s00467-025-07103-7

**Published:** 2026-01-19

**Authors:** Rachele Spagnol, Edoardo La Porta, Daniela Zugna, Silvia Consolo, Isabella Guzzo, Bruno Minale, Mario Giordano, Bruno Gianoglio, Carmela Errichiello, Ciro Corrado, Roberto Chimenz, Irene Alberici, Ester Conversano, Eleonora Guasti, Chiara Paccagnella, Marta Ferrecchi, Enrico Vidal, Enrico Verrina

**Affiliations:** 1 Pediatric Nephrology Unit, Department of Women and Children Health, University-Hospital of Padua, Padua, Italy; 2 Division of Nephrology, Dialysis and Transplantation, IRCCS Istituto Giannina Gaslini, Genoa, Italy; 3 Department of Medical Sciences, University of Turin, CPO-Piemonte, Turin, Italy; 4 Pediatric Nephrology, Dialysis and Transplant Unit, Fondazione IRCCS Ca’ Granda, Ospedale Maggiore Policlinico, Milan, Italy; 5 Division of Nephrology, Ospedale Pediatrico Bambino Gesù IRCCS, Rome, Italy; 6 Division of Nephrology and Dialysis, Pediatric Hospital Santobono, Naples, Italy; 7 Pediatric Nephrology and Dialysis Unit, Pediatric Hospital “Giovanni XXIII”, Bari, Italy; 8 Pediatric Nephrology Dialysis and Transplantation, University Hospital “Città della Salute e della Scienza di Torino”, Turin, Italy; 9 Nephrology and Dialysis Unit, Meyer Children’s Hospital IRCCS, Florence, Italy; 10 Pediatric Nephrology Unit, “G. Di Cristina” Hospital, Palermo, Italy; 11 Pediatric Nephrology and Dialysis Unit, University Hospital “G. Martino”, Messina, Italy; 12 Pediatric Nephrology and Dialysis Unit, IRCCS Azienda Ospedaliero-Universitaria di Bologna, Bologna, Italy; 13 DINOGMI, University of Genoa, IRCCS Giannina Gaslini, Genoa, Italy; 14 Division of Nephrology and Dialysis, Department of Medicine, University of Verona, Verona, Italy; 15 Department of Medicine (DMED), University of Udine, Udine, Italy; 16 University of Padua, Department of Children and Women Health, Padua, Italy

**Keywords:** Chronic hemodialysis, Complication, Hospitalization, Pediatrics, Peritoneal dialysis

## Abstract

**Background:**

Children receiving kidney replacement therapy frequently face complications resulting in recurrent hospitalizations. This nationwide retrospective observational study, conducted using data from the Italian Registry of Pediatric Chronic Dialysis (IRPCD), aimed to compare hospitalization rates and causes between children treated with chronic peritoneal dialysis (PD) and hemodialysis (HD).

**Methods:**

The study included children (< 18 years) on chronic PD or HD recorded between January 2000 and December 2019. Hospitalizations were defined as admissions involving at least one overnight stay, excluding those for dialysis initiation or kidney transplantation. Hospitalization causes were categorized as infectious and non-infectious dialysis-related complications, other infections, non-infectious conditions, diagnostic procedures, and complications related to kidney failure.

**Results:**

A total of 847 dialysis patients (493 on PD, 354 on HD) were included. Among 813 patients, 420 (51.7%) were hospitalized, with PD accounting for 72.9% at the first hospitalization. Dialysis-related infections were the most common cause (24.3%), particularly in PD patients, followed by non-infectious medical conditions (17.3%) and kidney failure-related complications (14.9%). Cox modeling indicated a lower risk of hospitalization for HD compared to PD (aHR 0.75 [95%CI 0.65–0.87]), with HD showing a protective effect over time. HD patients also had a lower likelihood of treatment changes after one year compared to PD (aHR 0.29 [95%CI 0.10–0.81]).

**Conclusions:**

This study highlights the significant burden of hospitalization among children on chronic dialysis, with PD patients experiencing higher risks over time compared to HD. These findings underscore the need for targeted strategies to mitigate hospitalization risks in pediatric dialysis populations.

**Graphical abstract:**

**Figure:**
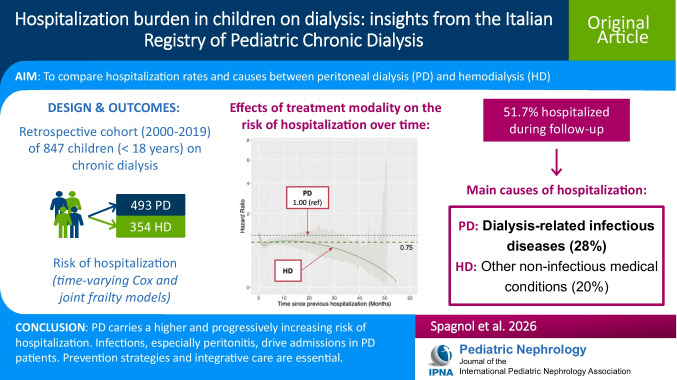
A higher resolution version of the Graphical abstract is available as Supplementary information

**Supplementary Information:**

The online version contains supplementary material available at 10.1007/s00467-025-07103-7.

## Introduction

In children with kidney failure, dialysis represents the most frequently employed kidney replacement therapy (KRT) and serves as a crucial bridge to kidney transplantation (KTx) [1–3]. However, dialysis is often complicated by numerous infectious and non-infectious events, as well as sequelae of chronic kidney disease (CKD), which increase the risk of hospitalization [4–6]. In some cases, these complications may even preclude KTx, leading to patient death [7, 8]. Frequent hospitalizations, while necessary for managing complications, are associated with increased morbidity, mortality and a significant reduction in quality of life [9].

In recent years, attention has focused on comparing the risks and outcomes associated with peritoneal dialysis (PD) and hemodialysis (HD), particularly in terms of mortality [7]. Pediatric European data demonstrated similar survival rates for PD and HD patients up to 24 months of age, but patients on long-term HD exhibited a lower cumulative hazard ratio (CHR) for mortality (CHR 0.22 [95%CI 0.16–﻿0.29]) [10]. In adults, mortality risk among PD patients increases over time, primarily because of dialysis-related complications, including recurrent peritonitis and peritoneal membrane damage, as well as the progressive loss of residual kidney function, which also occurs in HD patients [11].

Hospitalization rates, which can be seen as an important measure of morbidity and care quality, have been less consistently analyzed [12, 13]. US data showed that pediatric patients on dialysis experience significantly higher hospitalization rates than the general pediatric population, with conflicting findings regarding differences between PD and HD [14, 15]. A multicenter European study reported 0.35 and 0.46 admissions per 100 patient-days at risk in children treated with PD or HD, respectively, with no statistically significant difference (p = 0.19) [12]. Despite similar rates, the study highlighted distinct causes of hospitalization. Access-related infections, such as peritonitis, were the most common cause in PD patients, representing 24% of admissions. In contrast, non-infectious access-related complications, such as vascular access dysfunction, were the leading cause in HD patients, accounting for 19% of hospitalizations [12].

While previous studies have provided valuable insights, there is still a lack of large nationwide investigations with long-term follow-up to better clarify hospitalization trends and complications in pediatric dialysis. In this nationwide retrospective study, we used data from the Italian Registry of Pediatric Chronic Dialysis (IRPCD) to fill these gaps and to quantify and compare hospitalization rates and causes between PD and HD, focusing on their changes over time. As a secondary aim, we also assessed complications leading to hospitalization and the likelihood of switching dialysis modality, to better understand the long-term management of these patients.

Based on these considerations, we hypothesized that children on chronic PD would experience a higher and progressively increasing risk of hospitalization over time compared to those on HD, primarily driven by dialysis-related infections.

## Materials and methods

### Data collection

We conducted a retrospective analysis of the records of all patients who started dialysis before the age of 18 years, over a 20-year period (from January 2000 to December 2019). This data was collected by the IRPCD, a nationwide, population-based network that includes all 12 pediatric dialysis centers in Italy. Data were gathered via a web-based platform. Patients were eligible if they had been on chronic dialysis for the first time for at least 90 days; hence, patients with a previous transplant who returned to dialysis were excluded. Baseline information included age, gender, underlying kidney disease, comorbidities, dialysis modality, and age and calendar year when treatment was started. Comorbidities were defined as chronic medical conditions present at dialysis initiation and systematically recorded in the registry (congenital malformation syndrome, cardiovascular disease, neurodevelopmental delay, chronic inflammatory, pulmonary or intestinal disease, liver failure, diabetes mellitus, chromosomal anomaly, or neoplasia); each condition was counted separately, and patients were categorized according to the number of comorbidities (0, 1, 2, or ≥ 3). Conditions developing later during follow-up were not captured. All patients included in the registry provided informed consent, in accordance with ethical guidelines.

The primary outcome was the risk of hospitalization, analyzed as a recurrent event, in children on chronic PD and HD with additional descriptive analyses of hospitalization causes. The secondary outcome was the probability of switching dialysis modality during follow-up.

### Hospitalization

Hospitalization was defined as any admission that required at least one overnight stay. Hospitalizations were recorded from the time dialysis started until the study's end (December 31, 2019), KTx, or the patient's death — whichever occurred first. Hospitalizations related to dialysis initiation and KTx were excluded from the analysis. Collected data included: admission and discharge dates, type of admission, diagnosis for each hospitalization, length of stay (days), and outcome (recovery, change of treatment, or death).

Hospitalization causes were categorized into the following groups:

i. Dialysis-related infectious diseases (e.g. peritonitis or exit-site infections in PD, vascular access infections in HD);
ii. Dialysis-related non-infectious conditions (e.g. catheter displacement or leakage in PD, mechanical complications or thrombosis of catheter in HD);
iii. Other infectious conditions (e.g. respiratory, urinary tract or central nervous system infections);
iv. Other non-infectious medical conditions (acute admissions for non-infectious problems not directly related to CKD, e.g. seizures);
v. Diagnostic tests (e.g. peritoneal equilibration tests, diagnostic imaging requiring admission, fistulography, listing for KTx);
vi. Surgical procedures (e.g. nephrectomy, appendicectomy, percutaneous endoscopic gastrostomy placement);
vii. Kidney failure-related complications (e.g. anemia, hyperparathyroidism, hypertension, dyselectrolitemia);
viii. Unknown causes.

A descriptive analysis was also performed to explore whether the causes of hospitalization changed over time during follow-up.

### Statistical analysis

Continuous variables were expressed as median and interquartile range (IQR 25th-75th percentile) and categorical variables were presented as frequencies (N, %). The risk of hospitalization due to complications was modelled as a recurrent event over time considering KTx and death as terminal events. A structural joint frailty model was used to estimate the effect of the time-varying treatment (HD vs. PD) on the risk of hospitalization [16]. Patients who switched dialysis modality during follow-up contributed person-time to each modality until the date of switch, and hospitalizations were attributed to the modality in use at the time of admission. This approach aimed to estimate the short-term average causal effect of the treatment by comparing the probabilities of hospitalizations for complications in HD versus PD in each hospitalization episode. Two joint models for the hazards of recurrent event and the terminal event for each subject according to the treatment pattern were defined. Royston-Parmar survival model was used to model the hazard functions of the recurrent and terminal events [17]. Specifically, each model was characterized by time-varying regression coefficients for the treatment and a common individual frailty term by which the correlation between the two hazards was considered. The associations were expressed as adjusted hazard ratios (aHR) with their 95% confidence intervals (CI). Appropriate degrees of freedom for the baseline hazard functions were selected, and time-dependent treatment coefficients were derived by including an interaction term between treatment and time, modelled by a restricted cubic spline.

As secondary analysis, the risk of change of the first dialytic treatment was modelled considering KTx and death as competing events. Follow-up of patients was defined from the time dialysis started to the first change of treatment, KTx, patient’s death or study’s end, whichever occurred first. Non-parametric cumulative incidences were calculated, and potential differences were tested by Gray’s test, which is specifically designed to compare cumulative incidence functions in the presence of competing risks [18]. A Cox model was performed to estimate the effect of the treatment (HD vs. PD) on the risk of treatment change. Since the proportionality assumption was not satisfied, two interactions between treatment and time and age and time, respectively, were introduced into the model to allow the effect of treatment and age to change over time. In both analyses the following potential confounders were considered: gender, age and year when treatment began, number of comorbidities and primary kidney disease. All models were therefore adjusted for these variables.

The study is reported in accordance with the STROBE guidelines for observational cohort studies.

## Results

### Patient characteristics

**Fig. 1:**
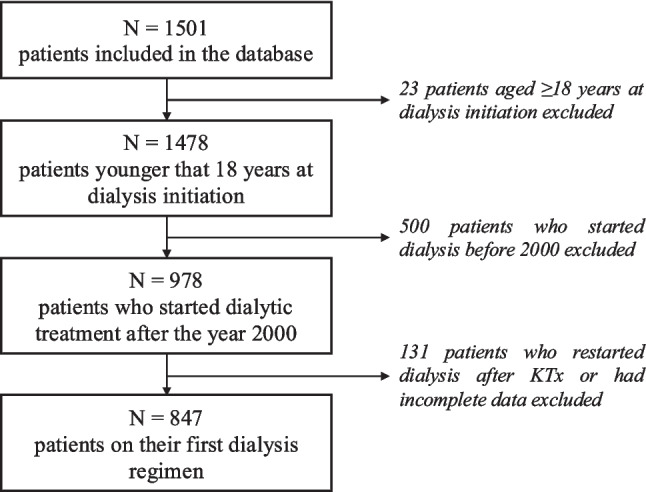
Flowchart of patient selection. The diagram shows the number of patients assessed for eligibility, the reasons for exclusion, and the final cohort of 847 children included in the analysis

The IRPCD included a total of 1487 patients under the age of 18 years at the time of KRT initiation. Of these, 978 fell within the observation period for this analysis. As shown in Fig. 1, the final sample comprised 847 patients, whose characteristics are described in Table 1. Of these, 493 (58.2%) were receiving PD and 354 (41.8%) HD.

**Table 1:** Characteristics of patients by treatment regimen (N = 847) (N, % for categorical variables, median (IQR) for continuous variables)

Population *N* = 847	PD *N* = 493 *(58.2)*	HD *N* = 354 *(41.8)*
Gender		
Male	199 *(40.4)*	160 *(45.2)*
Female	294 *(59.6)*	194 *(54.8)*
N° of comorbidities		
0	396 *(80.3)*	302 *(85.3)*
1	73 *(14.8)*	47 *(13.3)*
2	21 *(4.3)*	4 *(1.1)*
3	3 *(0.6)*	1 *(0.3)*
Cause of CKD		
CAKUT	194 *(41.6)*	102* (32.0)*
Cystic kidney disease	73 *(15.7)*	44 *(13.8)*
Glomerulonephritis	75 *(16.1)*	81 *(25.4)*
HUS	21 *(4.5)*	9 *(2.8)*
Hereditary nephropathy	27 *(5.8)*	25 *(7.8)*
Ischemic kidney failure	16 *(3.4)*	2 *(0.6)*
Metabolic disorders	11 *(2.4)*	13 *(4.1)*
Miscellaneous	48 *(10.3)*	31 *(9.7)*
Missing	89	58
Age at the beginning of treatment	5.0 (1.0–10.7)	13.0 (9.6–15.3)
Calendar year at beginning of treatment	2007 (03–13)	2011 (06–16)

Data indicated that patients on PD began treatment at a lower median age (5.0 years [IQR 1.0–10.7]) compared to those on HD (13.0 years [IQR 0.6–15.3]). Similarly, the initiation of PD occurred at an earlier median calendar year (2007 [IQR 2003–2013]) compared to HD (2011 [IQR 2006–2016]). The gender distribution showed a higher prevalence of females (58%) and it remained consistent across both dialysis modalities. 149 patients (17.6%) had at least one comorbidity, with a higher percentage of patients with more than one comorbidity in the PD compared to the HD group (19.7% vs. 14.7%, respectively) (Table 1). The leading cause of kidney failure in both groups was congenital anomalies of the kidney and urinary tract (CAKUT), with a prevalence of 41.6% in patients on PD and 32% in those on HD. Glomerulonephritis was the second most common cause in both groups, with a significantly higher prevalence in the HD group (25.4%) compared to the PD group (16.1%) (Table 1).

### Causes and risk of hospitalization

Complete data on hospitalizations were available for 813 out of 847 patients included. Among these, 420 required at least one hospitalization during the entire dialysis course, with a higher prevalence in the PD (N = 306/493 [62%]) compared to the HD cohort (N = 114/354 [32%]) after dialysis initiation. Considering patients hospitalized at least one time, the median number of hospitalizations per patient was 2 (IQR 1–4). The median length of hospitalization was 4 days in both the HD (IQR 1.4–10.7) and the PD group (IQR 1.3–8.8) and the median number of hospitalizations per center was 15.5 (IQR 1.7–129.5).

**Fig. 2:**
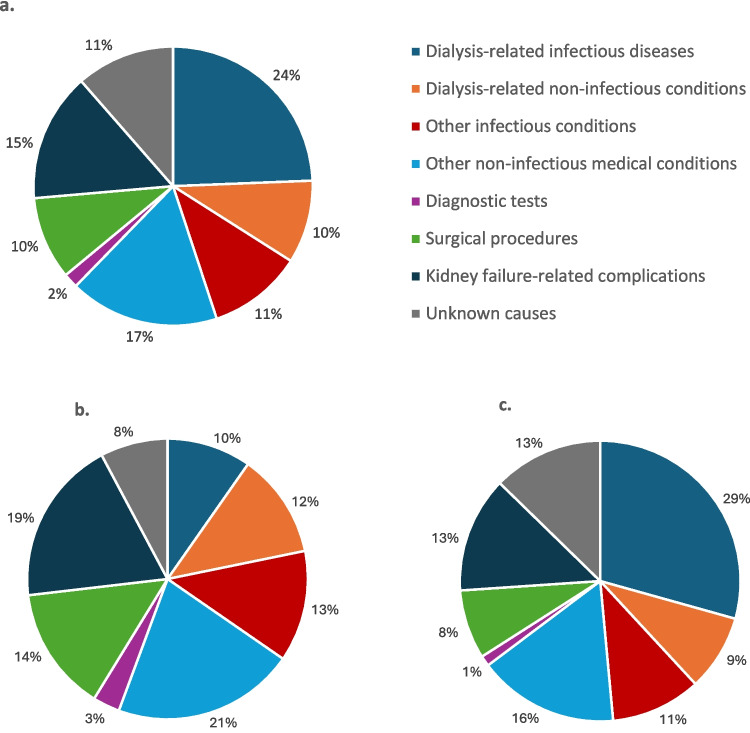
Distribution of the causes of hospitalization across the entire study population (**a**), in HD (**b**) and in PD (**c**)

The distribution of the hospitalization causes is reported in Fig. 2. Across the entire study population (Fig. 2a), the most frequent causes of hospitalization were dialysis-related infectious diseases (24.3%), followed by other non-infectious medical conditions (17.3%), and kidney failure-related complications (14.9%). In patients on HD the primary causes of hospitalization were non-infectious medical conditions (21.0%), and complications related to kidney failure (19.0%), followed by surgical procedures (14.3%) (Fig. 2b). In children receiving PD, a predominance of dialysis-related infectious conditions was found (29.3%), followed by other non-infectious medical conditions (16.2%), and kidney failure-related complications (13.3%) (Fig. 2c).

**Fig. 3:**
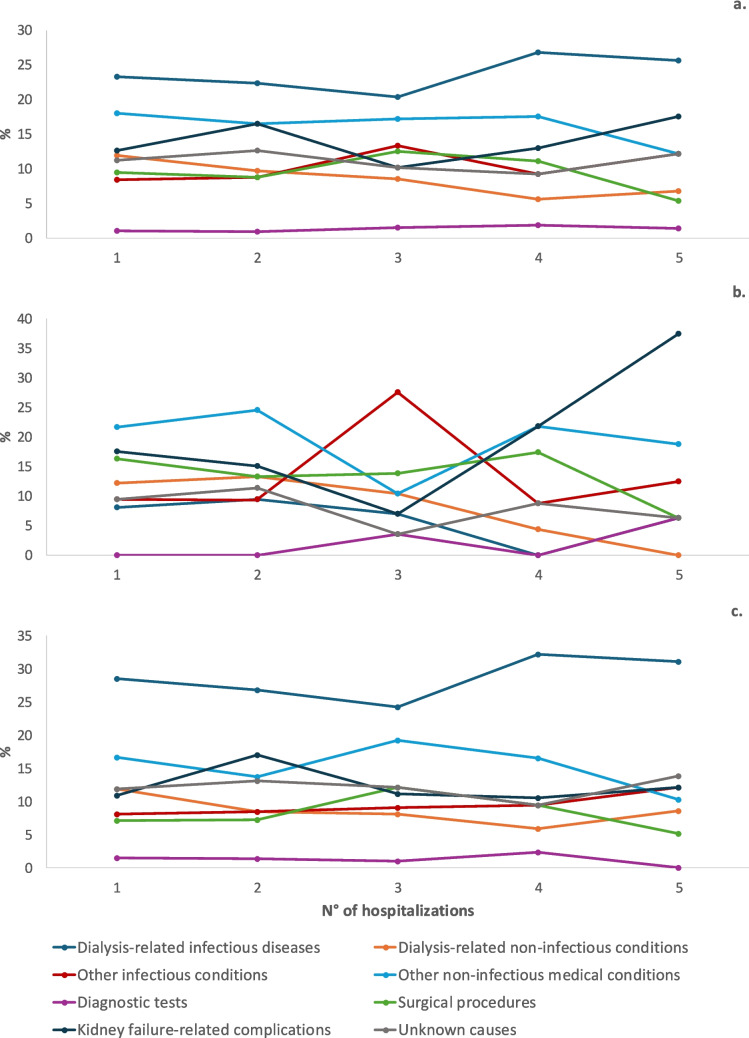
Frequency of each cause according to the cumulative number of hospitalizations across the entire study population (**a**), in HD (**b**) and in PD (**c**)

The frequency of each cause according to the order of hospitalization (first vs. subsequent admissions) and dialysis modality is reported in Fig. 3. The distribution of causes was similar between overall (Fig. 3a) and the PD group (Fig. 3c), with a predominance of dialysis-related infectious conditions in both initial and subsequent hospitalizations, followed by other non-infectious medical conditions. In the HD group (Fig. 3b), this distribution was less consistent: other non-infectious medical conditions were predominant in the first hospitalizations, while in subsequent admissions other infectious conditions and complications related to CKD stage V became more prominent, with the latter being particularly dominant in later hospitalizations.

**Fig. 4:**
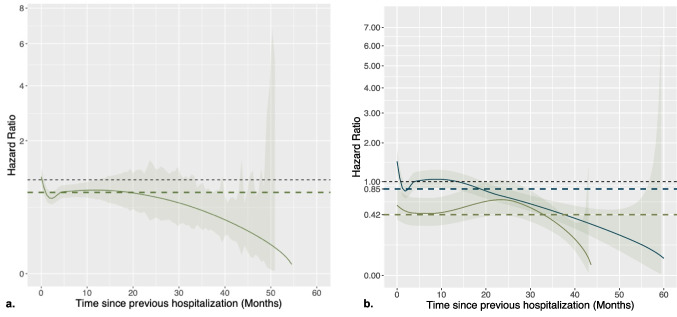
Panel **a** shows time-dependent hazard ratio (HR) of hospitalization for HD versus PD estimated by the structural joint frailty model (solid line), compared with the constant effect estimate from the Cox model (bold dashed line); the thin dashed line indicates the null effect (HR = 1). Panel **b** shows the decade-stratified analysis, with estimates for 2000–2009 (dark gray) and 2010–2020 (green)

We initially applied a Cox proportional hazards model to evaluate the risk of hospitalization, assuming a constant effect of dialysis modality over time. In this analysis, hemodialysis was associated with a lower risk of hospitalization compared to peritoneal dialysis (aHR 0.75, 95% CI 0.65–0.87). The model was subsequently extended to incorporate time-varying coefficients, allowing the effect of dialysis modality to vary during follow-up. The hazard ratio over time is shown in Fig. 4a. The model suggested a protective effect of HD compared to PD over time with respect to the risk of hospitalization. This effect became even more apparent when stratifying by decade, with a consistently higher and statistically significant risk for PD in 2010–2020 (aHR 0.42 [95% CI 0.31–0.56]) compared with the earlier period (< 2010, aHR 0.85 [95% CI 0.71–1.03]), as shown in Fig. 4b. Additionally, as shown in Table 2, this risk decreased with older age (aHR 0.95 [95%CI 0.93–﻿0.96), *p* < 0.001] and calendar year (aHR 0.96 [95%CI 0.95–0.97], *p* < 0.001) and increased with the number of comorbidities (aHR 1.19 [95%CI 1.05–﻿1.35], *p* = 0.006); no significant correlation was found with gender and underlying condition.

**Table 2:** Adjusted risk of hospitalization over time modelled as repeated event

	Adjusted HR (95%CI) of hospitalization over time modelled as repeated event	*p* value
Gender		
Male	1.00 (ref)	0.541
Female	0.95 (0.81; 1.11)	
Age (unit increase)	0.95 (0.93; 0.96)	< 0.001
Year (unit increase)	0.96 (0.95; 0.97)	< 0.001
#Comorbidities (unit increase)	1.19 (1.05; 1.35)	0.006
Disease		
CAKUT	1.00 (ref)	
Cystic kidney disease	1.15 (0.92; 1.43)	0.200
Glomerulonephritis	1.05 (0.84; 1.31)	0.661
Hereditary nephropathy	1.06 (0.74; 1.52)	0.759
HUS	1.02 (0.74; 1.39)	0.923
Ischemic kidney failure	1.10 (0.69; 1.74)	0.697
Metabolic disorders	1.09 (0.73; 1.62)	0.669
Miscellaneous	0.99 (0.77; 1.26)	0.907
Vasculitis	0.92(0.44; 1.95)	0.835

### Risk of switching modality

**Fig. 5:**
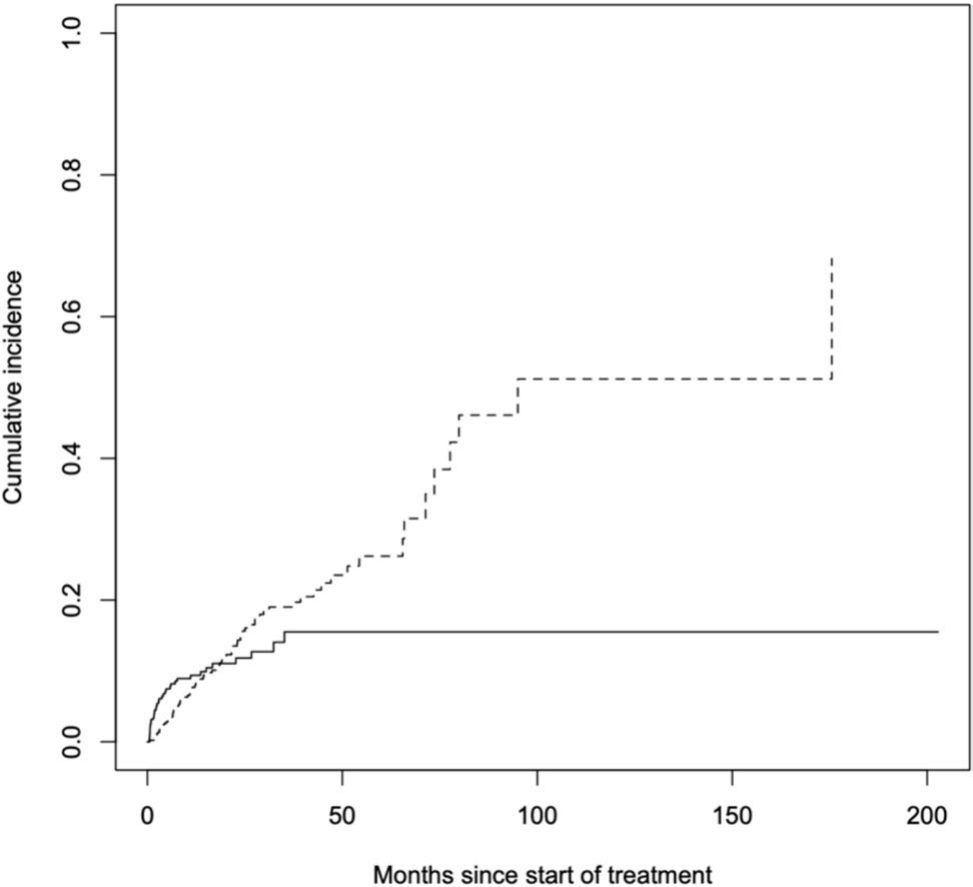
Risk of change of treatment over time for HD (solid line) and PD (dashed line)

As secondary analysis, 785 patients with complete data were included. Non-parametric cumulative incidence curves and Cox model were performed to evaluate the effect of the treatment (HD vs. PD) on the risk of changing dialysis modality. Our data showed that, compared with PD, children on HD had initially (within the first 60 days after the start) a higher risk of switching modality (aHR = 5.34, 95%CI 2.52–﻿11.32), probably reflecting patients who needed to start dialysis in an urgent and unplanned manner (Table 3, Fig. 5). On the contrary, patients on PD for more than one year had a higher chance of changing modality compared to those on HD (aHR = 0.29 [95%CI 0.10–﻿0.81]).

**Table 3:** Adjusted hazard ratios for change of treatment over time (*N* = 785)

		Adjusted HR (95% CI) with interaction between treatment and time to hospitalization
Gender		
Female		1.00 (ref)
Male		0.74 (0.45; 1.22)
Age (unit increase)		0.91 (0.85; 0.97)
Year (unit increase)		0.98 (0.95; 1.02)
#Comorbidities (unit increase)		1.17 (0.79; 1.73)
Disease		
CAKUT		1.00 (ref)
Cystic kidney disease		1.20 (0.58; 2.50)
Glomerulonephritis		1.55 (0.76; 3.18)
Hereditary nephropathy		1.41 (0.52; 3.77)
HUS		1.35 (0.45; 4.05)
Ischemic kidney failure		0.43 (0.09; 1.89)
Metabolic disorders		2.22 (0.73; 6.71)
Miscellaneous		1.42 (0.68; 2.98)
Vasculitis		8.86 (2.93; 26.78)
Treatment interval: 0–60 days	PD HD	1.00 (ref) 5.34 (2.52; 11.32)
Treatment interval: 60–180 days	PD HD	1.00 (ref) 1.48 (0.60; 3.65)
Treatment interval: 180–365 days	PD HD	1.00 (ref) 0.64 (0.23; 1.77)
Treatment interval: 365–730 days	PD HD	1.00 (ref) 0.29 (0.10; 0.81)
Treatment interval: 730 + days	PD HD	1.00 (ref) 0.43 (0.09; 2.04)

## Discussion

We hypothesized that children on chronic peritoneal dialysis would experience a higher and progressively increasing risk of hospitalization over time compared to those on hemodialysis, primarily driven by dialysis-related infections such as peritonitis. Our study provides a comprehensive evaluation of hospitalization rates among pediatric patients with CKD undergoing dialysis, comparing PD and HD. More than half of the children required hospitalization, with the first hospitalization being more frequent in PD than in HD. Dialysis-related infections were the primary cause in PD, whereas non-infectious conditions and CKD complications were more frequent in HD. Over time, HD demonstrated a lower hospitalization risk than PD, with risk decreasing with aging but increasing with comorbidities. Additionally, HD was associated with a higher early probability of modality switching, while prolonged PD led to an increased long-term risk.

In recent years, there has been a growing focus on understanding hospitalization risks in children undergoing chronic dialysis, given the profound implications for their quality of life and long-term survival. A 1996 study within the IRPCD found that children on PD had higher hospitalization rates and longer hospital stays than those on HD, particularly in younger age groups (below 5 years) [15]. In contrast more recent data from the 2020 USRDS report – although primarily referring to adults—showed no significant difference in overall hospitalization rates between PD and HD patients, with admission rates of 1.7 vs. 1.6 per patient per year, respectively [19].

Hospitalization not only reflects the clinical burden associated with kidney failure but also serves as a surrogate marker for the adequacy of care and the challenges of managing comorbidities in this vulnerable population. A recent prospective multicenter study from the European Pediatric Dialysis Working Group (EPDWG) provided valuable insights into hospitalization rates over a one-year period in pediatric patients across various European centers [12]. Our study addresses this issue on a nationwide scale, analyzing a large cohort of pediatric patients with CKD undergoing chronic dialysis over an extended 20-year observation period.

The study population’s characteristics align with expectations from the literature. Patients on PD were generally younger and had more comorbidities, reflecting its common use in small children. The causes of CKD followed established patterns [20], with CAKUT predominating in PD patients and glomerulonephritis being more frequent among those on HD. Notably, younger children with CAKUT who initiate PD often present with multiple comorbidities that substantially increase their medical complexity and susceptibility to infections. These observations are consistent with international registry data showing that nearly one third of pediatric PD patients have at least one non-kidney comorbidity, which is associated with higher hospitalization rates and lower survival [21]. This highlights the importance of a multidisciplinary approach, involving nephrologists and other specialists, to optimize care and reduce hospitalization burden in this fragile population.

In our analysis, dialysis-related infectious diseases were the leading cause of hospitalizations across the study population (23.3%), with a notably higher prevalence among patients on PD, where they accounted for 28.3% of admissions. These findings are consistent with those reported by Lafrance et al., who observed a 52% increased risk of infection-related hospitalizations in adult PD patients compared to HD patients [22]. Similarly, Bakkaloglu et al. identified access-related infections as the leading cause of hospitalization in pediatric patients on PD, accounting for 24% of cases [12]. In contrast, the causes of hospitalization among patients on HD showed a more balanced distribution, with non-infectious medical conditions being the most common (19.6%), followed by kidney failure-related complications (17.8%) and surgical procedures (13.4%). This variation underscores the diverse medical challenges faced by HD patients, compared to the predominance of infections in PD. Interestingly, Bakkaloglu et al. highlighted non-infectious complications of dialysis access as more frequent in HD patients (19%) [12], suggesting that differences in local practices, access management, and care strategies may account for this discrepancy [23–25]. These findings emphasize the need for a targeted approach in PD to mitigate infections, while HD management should address a wide range of potential complications.

Our study revealed significantly higher hospitalization rates among patients undergoing PD compared to those on HD. This differs from findings reported by the EPDWG, where no statistically significant differences in hospitalization rates between PD and HD patients were observed, possibly due to the shorter observation period [12]. A key strength of our research is the adjustment of hospitalization risk for confounding factors such as age, gender, comorbidities, and underlying conditions. Moreover, our analysis explored temporal trends, demonstrating consistently higher hospitalization risks for patients on PD over both short- and long-term periods. This risk may be related to the dialysis modality, with a higher likelihood of infectious complications in predominantly home-based techniques, such as peritoneal dialysis (PD), compared to hemodialysis (HD), which is usually performed in specialized centers with controlled environments.

Additionally, the frequent monitoring inherent to HD scheduling enables earlier detection and intervention for complications, potentially reducing the need for hospital admissions. Furthermore, our findings showed a higher likelihood of treatment modality switching among patients who had been on PD for at least one year, likely driven by the cumulative complications associated with this modality. These results align with observations by Heaf et al., who reported an early technique survival advantage for PD, followed by a greater tendency to transition to HD as complications arise over time [11]. Peritonitis in PD not only increases the immediate risk of hospitalization but also affects long-term outcomes by damaging the peritoneal membrane and reducing PD efficacy, ultimately contributing to the increased risk of switching to an alternative treatment modality [5, 26].

Our findings carry important implications for clinical practice. The high hospitalization rates observed underscore the need for targeted strategies to mitigate the burden of dialysis-related hospitalizations. The decade-stratified analysis suggests that recent technological improvements and practice changes may have contributed to more favorable outcomes for HD, while hospitalization rates remain higher for PD, underscoring the ongoing challenges of this modality. Given the elevated risk of hospitalization among patients on PD, priority should be placed on robust infection prevention measures, particularly for peritonitis. Comprehensive training and periodic retraining of medical and nursing staff, as well as caregivers, are essential, considering the home-based nature of PD management. Furthermore, the implementation of care bundles developed by the Standardizing Care to Improve Outcomes in Pediatric End-Stage Kidney Disease (SCOPE) Collaborative should be promoted and standardized across all pediatric dialysis centers [9, 27]. Average monthly peritonitis rates decreased from 0.53 (95%CI 0.37–﻿0.70) infections per patient-year pre-launch to 0.30 (95%CI 0.23–﻿0.43) at 84 months post-launch of bundles (*p* < 0.001) [28].

Given the high long-term risk of modality switching in PD patients, particularly among children undergoing prolonged dialysis, our findings support an "integrative care" approach. This strategy emphasizes initiating treatment with PD – especially in younger patients – and considering a transition to HD as PD-related complications become more frequent or technique failure occurs. Such an approach may help optimize long-term outcomes and reduce hospitalization burden in this population [10].

Our study has some limitations, primarily related to its retrospective design based on registry data. Information on some of the variables was incomplete or missing. Additionally, unmeasured confounders and changes in practice patterns over the 20-year observation period may have influenced the results. Moreover, the registry only records inpatient admissions and does not capture outpatient treatments. For example, some HD catheter infections may have been managed with intravenous antibiotics in an outpatient setting and therefore not included, potentially leading to an underestimation of hospitalization rates in HD patients. However, our study reports on data collected nationally through an established network including all 12 pediatric dialysis centers active in the country.

In conclusion, our study highlights the higher and progressively increasing risk of hospitalization associated with peritoneal dialysis compared to hemodialysis. Dialysis-related infections, especially peritonitis in PD patients, emerged as a major contributor to this elevated risk. These findings underscore the critical need for enhanced infection prevention measures and an integrative care approach to improve outcomes and quality of life for pediatric patients with kidney failure.

## Supplementary Information

Below is the link to the electronic supplementary material.

Graphical abstract (PPTX 562 KB)

## Data Availability

The datasets analyzed during the current study are not publicly available due to privacy regulations and data use agreements but are available from the corresponding author on reasonable request and with permission of the IRPCD Scientific Committee.

## References

[1] Harada R, Hamasaki S, Okuda R et al (2022) Epidemiology of pediatric chronic kidney disease/kidney failure: learning from registries and cohort studies. Pediatr Nephrol 37:1215–1229. 10.1007/s00467-021-05325-1 34091754 10.1007/s00467-021-05145-1

[2] Yu ED, Galbiati S, Munshi R et al (2022) Practice patterns and outcomes of maintenance dialysis in children <2 years of age: a report of the North American Pediatric Renal Trials and Collaborative Studies (NAPRTCS). Pediatr Nephrol 37:1117–1124. 10.1007/s00467-021-05234- 34648058 10.1007/s00467-021-05287-2

[3] Novljan G, Rus RR, Premru V et al (2016) Chronic hemodialysis in small children. Ther Apher Dial 20:302–307. 10.1111/1744-9987.12408 27312919 10.1111/1744-9987.12441

[4] Goldstein SL, Gerson AC, Goldman CW et al (2006) Quality of life for children with chronic kidney disease. Semin Nephrol 26:114–117. 10.1016/j.semnephrol.2006.01 16530604 10.1016/j.semnephrol.2005.09.004

[5] Vidal E (2018) Peritoneal dialysis and infants: further insights into a complicated relationship. Pediatr Nephrol 33:547–551. 10.1007/s00467-017-3857-3 10.1007/s00467-017-3857-3 29218436

[6] Teitelbaum I (2021) Peritoneal dialysis. N Engl J Med 385:1786–1795. 10.1056/NEJMra2100152 10.1056/NEJMra2100152 34731538

[7] Chesnaye NC, Schaefer F, Groothoff JW et al (2016) Mortality risk in European children with end-stage renal disease on dialysis. Kidney Int 89:1355–1362. 10.1016/j.kint.2016.01.016 27165828 10.1016/j.kint.2016.02.016

[8] Vidal E, van Stralen KJ, Chesnaye NC et al (2017) Infants requiring maintenance dialysis: outcomes of hemodialysis and peritoneal dialysis. Am J Kidney Dis 69:617–625. 10.1053/j.ajkd.2016.09.018 27955924 10.1053/j.ajkd.2016.09.024

[9] Atikel YÖ, Schmitt CP, Lévai E et al (2023) The effects of hospital and dialysis unit characteristics on hospitalizations for access-related complications among children on maintenance dialysis: a European, multicenter, observational, cross-sectional study. Pediatr Nephrol 38:2189–2198. 10.1007/s00467-023-05873-0 36595069 10.1007/s00467-022-05842-5

[10] Vidal E, Chesnaye NC, Paglialonga F et al (2018) A propensity-matched comparison of hard outcomes in children on chronic dialysis. Eur J Pediatr 177:117–124. 10.1007/s00431-017-3051-9 29143935 10.1007/s00431-017-3040-7

[11] Heaf JG, Løkkegaard H, Madsen M et al (2002) Initial survival advantage of peritoneal dialysis relative to haemodialysis. Nephrol Dial Transplant 17:112–117. 10.1093/ndt/17.1.112 11773473 10.1093/ndt/17.1.112

[12] Bakkaloğlu SA, Atikel JO, Schmitt CP et al (2023) Comparative analysis of hospitalizations among patients treated with hemodialysis and peritoneal dialysis in European pediatric nephrology centers: results from a prospective EPDWG/ESPN dialysis working group study. Clin Kidney J 17:747–756. 10.1093/ckj/sfad291 10.1093/ckj/sfad291 PMC10784969 38223336

[13] Bremer BA, McCauley CR, Wrona RM et al (1989) Quality of life in end-stage renal disease: a reexamination. Am J Kidney Dis 13:200–209. 10.1016/S0272-6386(89)80169-0 2493190 10.1016/s0272-6386(89)80053-8

[14] US Renal Data System (2018) Annual data report. Volume 1, Chapter 6. CKD among children and adolescents. https://www.ajkd.org/article/S0272-6386(18)31099-0/pdf. Accessed 10 Oct 2024

[15] Verrina E, Perfumo F, Zacchello G et al (1996) Comparison of patient hospitalization in chronic peritoneal dialysis and hemodialysis: a pediatric multicenter study. Perit Dial Int 16:S574–S577 8728274

[16] Liu L, Wolfe RA, Huang X (2004) Shared frailty models for recurrent events and a terminal event. Biometrics 60:747–756. 10.1111/j.0006-341X.2004.00225.x 15339298 10.1111/j.0006-341X.2004.00225.x

[17] Royston P, Parmar MK (2002) Flexible parametric proportional-hazards and proportional-odds models for censored survival data, with application to prognostic modelling and estimation of treatment effects. Stat Med 21:2175–2197. 10.1002/sim.1203 12210632 10.1002/sim.1203

[18] Gray RJ (1988) A class of K-sample tests for comparing the cumulative incidence of a competing risk. Ann Stat 16:1141–1154. 10.1214/aos/1176350951

[19] Johansen KL, Chertow GM, Foley RN et al (2021) US renal data system 2020 annual data report: epidemiology of kidney disease in the United States. Am J Kidney Dis 77:A7–A8. 10.1053/j.ajkd.2021.01.002 33752804 10.1053/j.ajkd.2021.01.002 PMC8148988

[20] National Kidney Foundation (2002) K/DOQI clinical practice guidelines for chronic kidney disease: evaluation, classification, and stratification. Am J Kidney Dis 39:S1–S266. 10.1053/ajkd.2002.30939 11904577

[21] Neu AM, Sander A, Borzych-Duzalka D et al (2012) Comorbidities in chronic pediatric peritoneal dialysis patients: a report of the International Pediatric Peritoneal Dialysis Network. Perit Dial Int 32:410–418. 10.3747/pdi.2012.00124 22859841 10.3747/pdi.2012.00124 PMC3524853

[22] Lafrance JP, Rahme E, Iqbal S et al (2012) Association of dialysis modality with risk for infection-related hospitalization: a propensity score-matched cohort analysis. Clin J Am Soc Nephrol 7:1598–1605. 10.2215/CJN.00440112 22904124 10.2215/CJN.00440112 PMC3463202

[23] Warady BA, Bakkaloğlu SA, Newland J et al (2012) Consensus guidelines for the prevention and treatment of catheter-related infections and peritonitis in pediatric patients receiving peritoneal dialysis: 2012 update. Perit Dial Int 32:S32–S86 22851742 10.3747/pdi.2011.00091 PMC3524923

[24] Coppolino G, Lucisano G, Bolignano D et al (2010) Acute cardiovascular complications of hemodialysis. Minerva Urol Nefrol 62:67–80 20424575

[25] Chouhani BA, Kabbali N, Chiba Bennani S et al (2022) Tunneled catheters in hemodialysis: indications and complications. J Med Vasc 47:87–93. 10.1016/j.jdmv.2021.11.005 35691668 10.1016/j.jdmv.2022.04.007

[26] Aksoy GK, Ekim M, Bakkaloğlu SA et al (2021) Evaluation of non-infectious complications of peritoneal dialysis in children: a multicenter study. Pediatr Nephrol 36:417–423. 10.1007/s00467-020-04722-9 32728843 10.1007/s00467-020-04719-9

[27] Neu AM, Miller MR, Stuart J et al (2014) Design of the standardizing care to improve outcomes in pediatric end stage renal disease collaborative. Pediatr Nephrol 29:1477–1484. 10.1007/s00467-014-2795-1 25055994 10.1007/s00467-014-2891-7

[28] Neu AM, Richardson T, De Souza HG et al (2021) Continued reduction in peritonitis rates in pediatric dialysis centers: results of the Standardizing Care to Improve Outcomes in Pediatric End Stage Renal Disease (SCOPE) Collaborative. Pediatr Nephrol 36:2383–2391. 10.1007/s00467-021-04924-0 33649895 10.1007/s00467-021-04924-0

