# Supplementary material for: Hospitalization burden in children on dialysis: insights from the Italian Registry of Pediatric Chronic Dialysis (IRPCD)
Source: Pediatr Nephrol. 2026 Jan 19;41(6):1785–95. doi: 10.1007/s00467-025-07103-7 (PMC13139294; doi:10.1007/s00467-025-07103-7)
Supplement: Supplementary file 1 — Graphical abstract (PPTX 562 KB) [file 467_2025_7103_MOESM1_ESM.pptx]

## Slide 1
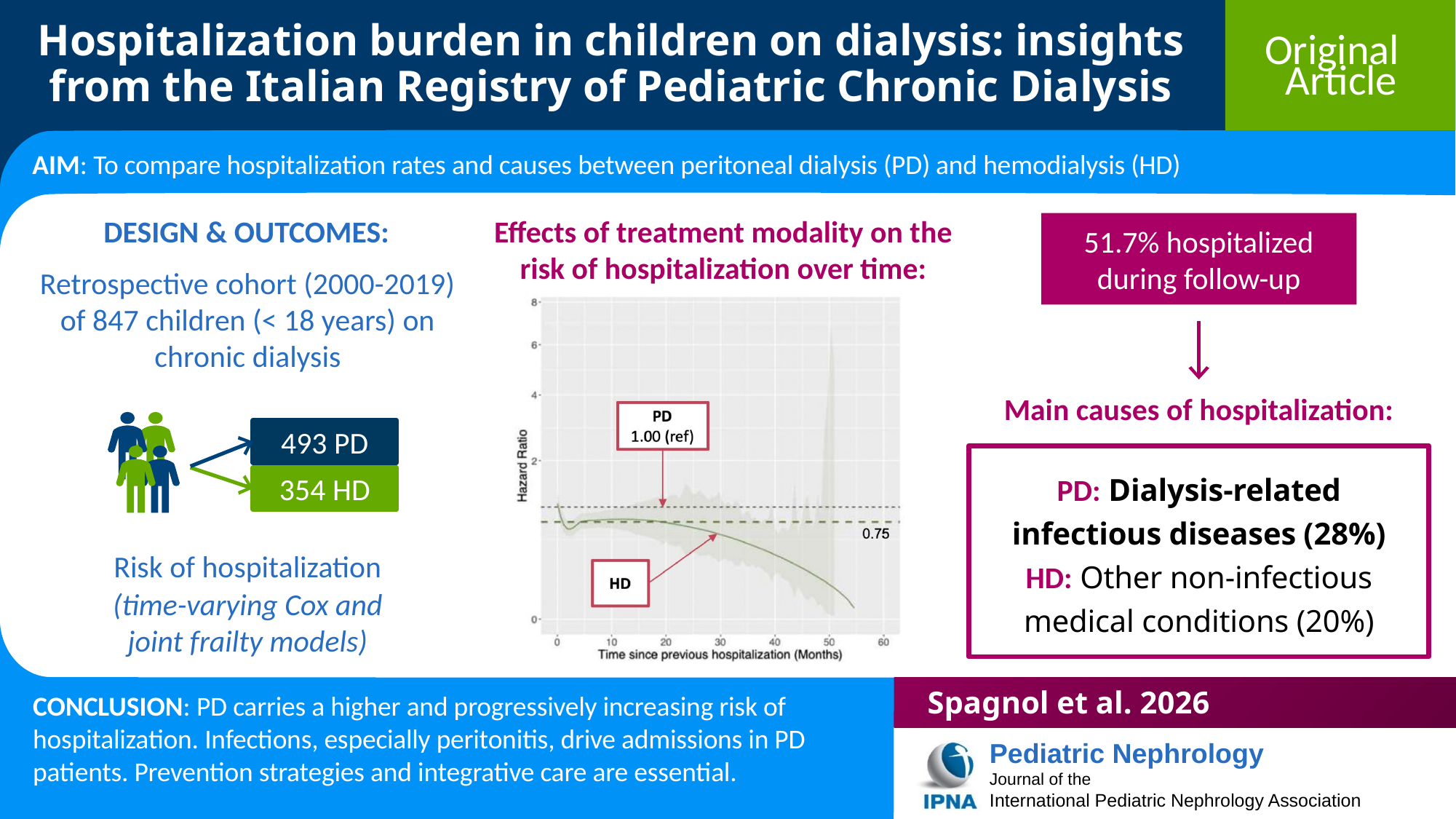

Hospitalization burden in children on dialysis: insights from the Italian Registry of Pediatric Chronic Dialysis
AIM: To compare hospitalization rates and causes between peritoneal dialysis (PD) and hemodialysis (HD)
DESIGN & OUTCOMES:
Effects of treatment modality on the risk of hospitalization over time:
51.7% hospitalized
during follow-up
Retrospective cohort (2000-2019) of 847 children (< 18 years) on chronic dialysis
Main causes of hospitalization:
493 PD
PD: Dialysis-related infectious diseases (28%)
HD: Other non-infectious medical conditions (20%)
354 HD
Risk of hospitalization
(time-varying Cox and joint frailty models)
Spagnol et al. 2026
CONCLUSION: PD carries a higher and progressively increasing risk of hospitalization. Infections, especially peritonitis, drive admissions in PD patients. Prevention strategies and integrative care are essential.
